# Risk factors associated with within-herd transmission of bovine leukemia virus on dairy farms in Japan

**DOI:** 10.1186/1746-6148-6-1

**Published:** 2010-01-07

**Authors:** Sota Kobayashi, Toshiyuki Tsutsui, Takehisa Yamamoto, Yoko Hayama, Ken-ichiro Kameyama, Misako Konishi, Kenji Murakami

**Affiliations:** 1 Epidemiological Research Team, National Institute of Animal Health, 3-1-5, Kannondai, Tsukuba-shi, Ibaraki, Japan; 2 Research Team for Viral Diseases, National Institute of Animal Health, 3-1-5, Kannondai, Tsukuba-shi, Ibaraki, Japan

## Abstract

**Background:**

Although several attempts have been made to control enzootic bovine leukosis (EBL) at the local level, a nationwide control program has not been implemented in Japan, except for passive surveillance. Effective control of EBL requires that the transmission routes of bovine leukemia virus (BLV) infection should be identified and intercepted based on scientific evidence. In this cross-sectional study, we examined the risk factors associated with within-herd transmission of BLV on infected dairy farms in Japan. Blood samples taken from 30 randomly selected adult cows at each of 139 dairy farms were tested by enzyme-linked immunosorbent assay (ELISA). Information on herd management was collected using a structured questionnaire.

**Results:**

Infected farms were defined as those with more than one ELISA-positive animal and accounted for 110 (79.1%) of the 139 farms in the study. Completed questionnaires obtained from 90 of these 110 farms were used for statistical analysis. Seroprevalence, which was defined as the proportions of animals that tested positive out of all animals tested on the farm, was 17.1%, 48.1%, and 68.5% for the 25th, 50th, and 75th percentiles, respectively. A mixed logistic regression analysis implicated a loose housing system, dehorning, and a large number of horseflies in summer as risk factors (coefficient = 0.71, 1.11, and 0.82; p = 0.03, < 0.01, and 0.01, respectively) and feeding of colostrum to newborn calves from their dams as a protective factor (coefficient = -1.11, p = 0.03) against within-farm transmission of BLV on infected farms.

**Conclusion:**

Control of EBL in infected dairy farms in Japan will be improved by focusing particularly on these risk and protective factors.

## Background

*Bovine leukemia virus *(BLV), a retrovirus of the family *Retroviridae*, is the causative agent of enzootic bovine leucosis (EBL). Approximately 30% of cattle infected with BLV have persistent lymphocytosis, and 1-5% develop B-cell lymphosarcoma [1]. With a worldwide distribution, EBL is listed by the World Organization for Animal Health as a disease of importance to international trade [2] and is included in the national eradication program in Australia and some member states of the European Union (EU), several of which have recently eliminated the disease [3,4]. In contrast, 89% of dairy herds in the United States are reported to be infected with BLV [5], and the annual economic loss due to EBL is estimated at $525 million in decreased milk yield [6]. Although EBL causes serious economic damage in the dairy industry [7], thus far only regional voluntary control programs have been implemented in the United States [8].

In Japan, EBL is listed as a notifiable disease, but no nationwide control programs have been established. According to animal health statistics, EBL was reported in 159 cattle at 157 farms in 2000 and in 838 cattle at 677 farms in 2007. These data suggest that EBL has been gradually spreading in Japan.

BLV is present in the circulating peripheral blood lymphocytes of infected cattle, and horizontal transmission of the virus occurs via infected blood often as a result of unhygienic farm practices such as the use of plastic sleeves to perform rectal palpation and the same needle for vaccination of more than one animal [9-11] or unhygienic dehorning practices [11-13]. Furthermore, infected lymphocytes may also be transmitted mechanically by hematophagous insects such as horseflies [14-16]. BLV transmission has also been reported through physical contact between infected and uninfected cattle [17,18]. In addition, since BLV and its antibodies are present in the colostrum and normal milk of infected cattle, BLV transmission from infected cows to calves via infected milk is negligible [19].

These transmission routes should be considered when designing preventive measures against EBL at the farm level. In addition, it may be important to determine which transmission routes are the most important for each farm. It is therefore essential to identify the risk factors associated with BLV transmission in the production systems targeted for disease control. However, no such epidemiological approach for BLV infection has been attempted in Japan. Therefore, the objective of this study was to clarify herd management factors associated with within-herd transmission of BLV and to facilitate a more rational and efficient approach for controlling BLV transmission on dairy farms in Japan.

## Results

Results of serological tests showed that 110 (79.1%) of 139 dairy farms were infected with BLV. Statistical analyses were then performed for 90 of these farms (81.8%), for which complete data were obtained from the questionnaires.

**Figure 1:**
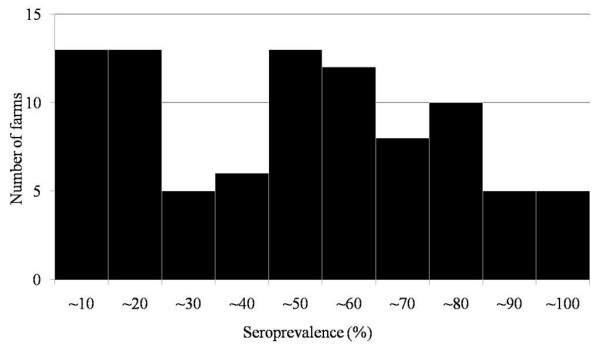
**Distribution of BLV-infected farms classified by seroprevalence (n = 90)**. Seroprevalence on infected farms at the 25th, 50th (median), and 75th percentiles was 17.1%, 48.1%, and 68.5%, respectively. Visual inspection of this histogram and the Shapiro--Wilk test confirmed that seroprevalence was not normally distributed (p < 0.002).

Seroprevalence at infected farms at the 25th, 50th (median), and 75th percentiles was 17.1%, 48.1%, and 68.5%, respectively (Figure 1 and Table 1). Visual inspection of Figure 1 and the Shapiro-Wilk test confirmed that seroprevalence was not normally distributed (p < 0.002). Univariate test results showed that cattle housing conditions, availability of own grazing area, the presence of horseflies in summer, dehorning, the use of a plastic sleeve for rectal palpation, not changing needles between animals during herd vaccination, and colostrum feeding were factors possibly associated with seroprevalence (p < 0.15, Table 1), whereas herd size (p = 0.51) and cattle replacement (p = 0.29) were not associated with it.

**Table 1:** Crude univariate analyses between seroprevalence of BVL and farm factors in seven prefectures in Japan

Variable and level	Number of herds	Median (IQR^1^)	**P**^2^
Herd size			
< 30 head	35	43.3 (13.3, 60.0)	0.514
30--49 head	25	53.33 (16.7, 63.3)	
≥ 50 head	30	50.00 (26.7, 73.3)	
Cattle introduced into the herd within one year			
No	11	46.7 (27.3, 60.0)	0.286
Yes, self-bred cows only	33	52.2 (25.0, 78.0)	
Yes, including purchased cows	46	45.0 (13.3, 65.0)	
Housing conditions			
Tie housing	68	41.9 (13.3, 62.5)	0.001*
Loose housing	22	65.0 (52.2, 78.3)	
Availability of own grazing area			
Yes	65	43.3 (13.8, 63.3)	0.048*
No	25	52.2 (33.6, 78.0)	
Presence of horseflies in summer			
Never to seldom	28	26.7 (13.3, 51.8)	0.002*
Sometimes to often	29	47.6 (15.8, 65.0)	
Very high	33	63.3 (44.4, 83.0)	
Animal dehorning			
Yes	58	60.0 (39.2, 73.3)	< 0.001*
No	32	20.0 (8.8, 43.0)	
Plastic sleeve used for rectal palpation			
One sleeve per cow	73	43.3 (14.5, 68.3)	0.012*
One sleeve for more than one cow	17	60.0 (47.8, 75.0)	
Needle used for vaccination			
One needle per cow	86	46.7 (16.7, 65.5)	0.100*
One needle for more than one cow	4	64.4 (50.5, 91.7)	
Colostrum feeding			
No	7	76.7 (60.0, 95.7)	0.003*
From dam to calves	63	42.1 (13.3, 60.0)	
Pooled	20	50.0 (36.7, 75.8)	

Overall	90	48.1 (17.1, 68.5)	

^1^Interquartile range

^2^Mann--Whitney U-test for two-level variables or Kruskal--Wallis test for all others

*Incorporated into multivariate model

Results of the Mann--Whitney U-test or the Kruskal--Wallis test showed that cattle housing conditions, availability of own grazing area, presence of horseflies in summer, dehorning, use of a plastic sleeve for rectal palpation, not changing needles between animals during herd vaccination, and colostrum feeding were possibly associated with seroprevalence (p < 0.15). These seven variables were incorporated in the multivariate model.

Starting with the seven variables with p values < 0.15 in the univariate analyses, the final model was obtained with four variables with the smallest value of the Akaike information criterion (AIC) (Table 2). A loose housing system, dehorning, and the observable presence of horseflies were associated with increases in seroprevalence (coefficient = 0.71, 1.11, 0.82; p = 0.03, < 0.01, 0.01). These were considered as risk factors that facilitated the within-farm transmission of BLV. In contrast, colostrum feeding of calves from their dams was considered to be a protective factor that suppressed the within-farm transmission of the virus (coefficient = -1.11, p = 0.03). No biologically plausible two-way interactions between the remaining factors were observed in the final model.

**Table 2:** Final logistic regression model with random herd effect for logit-transformed seroprevalence of BLV in Japan

Variable Level	**β**^1^	**SE**^2^	z-value	P of z-value
Intercept	-0.36	0.59	-0.62	0.54
Housing conditions				
Tied system	Ref.^3^			
Loose system	0.71	0.316	2.23	0.03
Animal dehorning				
No	Ref.			
Yes	1.11	0.302	3.66	0.0002
Presence of horseflies in summer				
Never or seldom	Ref.			
Sometimes or often	-0.24	0.341	-0.70	0.49
Very high	0.82	0.321	2.56	0.01
Colostrum feeding				
No	Ref.			
From dam to calves	-1.11	0.52	-2.13	0.03
Pooled	-0.90	0.55	-1.65	0.10

^1^estimated coefficients, ^2^standard error for the coefficient, ^3^reference category

Standard deviation in mixing distribution = 1.054, Standard error = 0.099

Starting from the full model with seven variables selected by univariate analyses, the best model was constructed on the basis of AIC. The best model with the smallest AIC included housing system, dehorning, observable presence of horseflies, and direct colostrum feeding. The coefficients (β values) indicate that loose housing, dehorning, and observation of a large number of horseflies in summer were positively associated with seroprevalence on infected farms (β values > 0, p values < 0.05). In contrast, feeding of colostrum was negatively associated with seroprevalence in the infected farms (β = -1.11, p = 0.03)

## Discussion

This is the first epidemiological study to assess herd management factors related to the seroprevalence of BLV on dairy farms in Japan. Based on our mixed logistic model with a random herd effect, the significant factors were found to be cattle housing conditions, the presence of horseflies in summer, dehorning practices, and colostrum feeding.

With regard to housing conditions, a loose housing system was found to be positively associated with seroprevalence compared with a tied housing system (p = 0.03). A possible explanation may be that direct contact between infected and uninfected cattle is facilitated in loose housing systems such as free stalls and barns. Furthermore, the chances of indirect contact between uninfected and infected cattle may be relatively high in a loose housing system because cattle randomly move about the barn or paddock and are not always handled in the same manner.

Dehorning is a practice in daily herd management that can reduce the risk of injury from horn-butting in farmers and in cattle during herd conflict. This practice was found to be positively associated with seroprevalence compared with farms without it (p < 0.01). A potential explanation for this result is the unhygienic procedure of dehorning, which is a risk factor for BLV transmission. It was previously reported that calves dehorned with contaminated dehorning apparatus were more likely to be infected than those that had not been dehorned [11-13]. Farmers often leave the dehorned heifers without hemostasis by cautery (personal communications with clinical veterinarians). Although we did not include a question related to the treatment of dehorned cattle in the questionnaire, the information obtained from clinical veterinarians supported our result.

With regard to the presence of horseflies in summer, a response of "very high" was positively associated with seroprevalence compared with a response of "never to seldom" (p = 0.01); however, there was no significant difference between "sometimes or often" and "never to seldom" (p = 0.49). BLV transmission by hematophagous insects has been reported [14-16]. In this study, we did not measure horsefly populations quantitatively on each farm. However, given the effect of the response of "very high" in increasing seroprevalence, our findings support the results of previous studies and suggest more quantitative study in future.

In the present study, colostrum feeding of calves from their dams resulted in a decrease in within-farm seroprevalence (coefficient = -1.11, p = 0.03). Because colostrum contains BLV and its antibodies, ingestion of colostrum from infected dams reduces the risk of BLV infection during the weaning period in calves [20-25]. Our result is consistent with previous studies, although there were only seven farms in our study without colostrum feeding, the reference category. In addition, despite marginal significance, pooled colostrum feeding on a farm had a negative impact on seroprevalence (coefficient = -0.90, p = 0.10). Therefore, colostrum feeding could be an effective way to reduce the prevalence of BLV in a herd. The effect of optimal conditions for the treatment of pooled colostrum, such as heating and freezing on the farm, should be evaluated in future studies.

Because this study had a cross-sectional design, it was difficult to elucidate definite relationships between seroprevalence and each risk or protective factor. However, in spite of this constraint, our results were consistent with previous studies and confirmed the important factors that should be controlled.

## Conclusions

Controlling BLV infection at Japanese dairy farms should focus on these factors. Future research is required to compare the influences of each factor responsible for within-herd transmission and to facilitate more rational prioritization of control measures.

## Methods

### Target population and sampling criteria

This cross-sectional study examined dairy farms located in 7 of the 47 prefectures in Japan. Each prefectural government sought participation from approximately 20 dairy farmers, and a total of 139 agreed to enroll. At each farm, the owner was asked for the blood sampling by the sample collectors who gave the minimum possible pain to the cattle, and only those who understood the study objectives were involved in this study. Each animal was firmly restrained before sampling to prevent unnecessary apprehension and pain to the animal and unexpected accidents to the sample collectors. Samples were then collected from the jugular or caudal vein. Blood samples were taken from 30 randomly selected adult cows (or all cows on farms of herd size < 30) at each farm between June and December, 2007. Separated sera were transferred to the National Institute of Animal Health (NIAH, Tsukuba, Japan) and stored at -20°C for serological tests. Since this study was a field survey, no ethical approval for the study was required by the animal care and ethical committee of National Institute of Animal Health.

### Questionnaire survey

Information regarding herd management was acquired at the time of blood sampling by local veterinary officers using a questionnaire prepared in advance [Additional file 1]. The questions inquired about cattle housing conditions, cow replacement, provision of own grazing area or free range on the farm, the presence of horseflies in summer, dehorning, the use of plastic sleeves for rectal palpation, change of needles used for herd vaccination, colostrum feeding, and general data on herd demography. Data from the completed questionnaires were stored and handled using commercially available spreadsheet software (Excel 2007; Microsoft Corp., Redmond, WA, USA).

### Serological testing

Each serum sample was serologically tested at NIAH using a commercial enzyme-linked immunosorbent assay (ELISA) kit (Bovine Leukosis Serum Screening ELISA; Pourquier, Montpellier, France). The procedures were performed according to the manufacturer's instructions. If the ELISA results were inconclusive, confirmation was sought using an agar gel precipitation test reported previously [26].

### Statistical analyses

In the present study, an infected farm was defined as a farm with more than one serologically positive cow. To analyze the risk factors associated with within-herd transmission of BLV, the subsequent modeling process targeted the seroprevalence of each infected farm as a response variable, which was defined as the proportion of cattle testing positive among all tested cattle on the farm.

To obtain a general overview of the association between seroprevalence and each herd management factor, the normality of the distribution of seroprevalence at infected farms was evaluated by visual inspection of the histogram and by the Shapiro-Wilk test, followed by univariate analysis. The Mann-Whitney U-test was used to analyze variables with two levels, and the Kruskal-Wallis test to analyze all other variables. Variables with a p value < 0.15 in these tests were used for multivariate model building. A generalized mixed linear model was used to evaluate the risk factors that influenced seroprevalence; the response variable being the logit of seroprevalence and the herd as a random effect. The model is described as follows:

p = number of positive animals/number of test animals,

where p represents the seroprevalence accounting for sample size, α is the model intercept, χ is fixed effects with p < 0.15 in the univariate analyses, β is its coefficient, RH is the random herd effect, and e is the binomially distributed residual term. The best model was constructed by a stepwise approach, observing the change in AIC of each model. The final model was obtained with the minimum AIC and p < 0.05 for the remaining fixed effects.

All statistical analyses were performed using R version 2.6.1 (R Development Core Team, 2007), and the glmmML package [27] was used for model building.

## Authors' contributions

SK participated in the design of the study, performed all the data handling and statistical analyses, and drafted the manuscript. TT participated in the design of the study and helped to draft the manuscript. TY and YH investigated the data analyses performed by SK. KK and MK performed serological diagnoses and the related laboratory work. KM conceived the study, participated in its design and coordination, and helped to draft the manuscript. All authors read and approved the final manuscript.

## Supplementary Material

Additional file 1 **Questionnaire used for the sero-epidemiological survey of the enzootic bovine leukosis in Japan, 2007**. The questions inquired about cattle housing conditions, cow replacement, provision of own grazing area or free range on the farm, the presence of horseflies in summer, dehorning, the use of plastic sleeves for rectal palpation, change of needles used for herd vaccination, colostrum feeding, and general data on herd demography. Click here for file
