# Supplementary material for: Risk factors associated with within-herd transmission of bovine leukemia virus on dairy farms in Japan
Source: BMC Vet Res. 2010 Jan 7;6:1. doi: 10.1186/1746-6148-6-1 (PMC2835688; doi:10.1186/1746-6148-6-1)
Supplement: Additional file 1 — Questionnaire used for the sero-epidemiological survey of the enzootic bovine leukosis in Japan, 2007. The questions inquired about cattle housing conditions, cow replacement, provision of own grazing area or free range on the farm, the presence of horseflies in summer, dehorning, the use of plastic sleeves for rectal palpation, change of needles used for herd vaccination, colostrum feeding, and general data on herd demography. [file 1746-6148-6-1-S1.DOC]

Date: _____________________

　Prefecture: ____________________

| EBL Survey Questionnaire |
| --- |
| 1. Farm owner:  Address: |
| 2. Herd size  Calf: Heifer: Adult: |
| 3. Animal introduction within one year  Yes / No  If yes, it was done:  with home-bred animal only / including purchased animal |
| 4. Cattle housing  Loose housing / Tie housing |
| 5. Availability of own grazing area  Yes / No |
| 6. Frequency to observe the horseflies in summer  None / Seldom / Sometimes / Often / Very high |
| 7. Conduct dehorning?  Yes / No |
| 8. Frequency of changing plastic sleeves for rectal palpation  One sleeve per cow / One sleeve for more than one cow |
| 9. Frequency of changing needles for vaccination  One needle per cow / One needle for more than one cow |
| 10. Colostrum feeding  Yes / No  If yes, it is done by:  pooled colostrum / colostrum from dam |
